# Liver steatosis, cardiac and renal fibrosis, and hypertension in overweight rats: Angiotensin-(3–4)-sensitive hepatocardiorenal syndrome

**DOI:** 10.1016/j.metop.2022.100176

**Published:** 2022-03-18

**Authors:** Thuany Crisóstomo, Marco A.E. Pardal, Simone A. Herdy, Humberto Muzi-Filho, Debora B. Mello, Christina M. Takiya, Rafael Luzes, Adalberto Vieyra

**Affiliations:** a Leopoldo de Meis Institute of Medical Biochemistry, Federal University of Rio de Janeiro, Rio de Janeiro, Brazil; b National Center of Structural Biology and Bioimaging/CENABIO, Federal University of Rio de Janeiro, Rio de Janeiro, Brazil; c Carlos Chagas Filho Institute of Biophysics, Federal University of Rio de Janeiro, Rio de Janeiro, Brazil; d Graduate Program in Translational Biomedicine/BIOTRANS, University of Grande Rio, Duque de Caxias, Brazil

**Keywords:** Hyperlipidic diet, Overweight rats, Arterial hypertension, Cardiac and renal fibrosis, Non-alcoholic liver steatosis, Hepatocardiorenal syndrome

## Abstract

Overweight/obesity is a growing pandemic that affects many organs and tissues. We have investigated whether a high-lipid diet provokes an imbalance between type 1 and type 2 angiotensin II (Ang II) receptors signaling, leading to liver alterations associated with cardiovascular and kidney disturbances. Chronic administration of a high-lipid diet can provoke hepatocardiorenal syndrome resulting from activation of the Ang II→type 1 receptor axis, which is entirely counteracted by Ang-(3–4), the allosteric enhancer of the Ang II→type 2 receptor pathway.

## Graphical abstract

**Figure:**
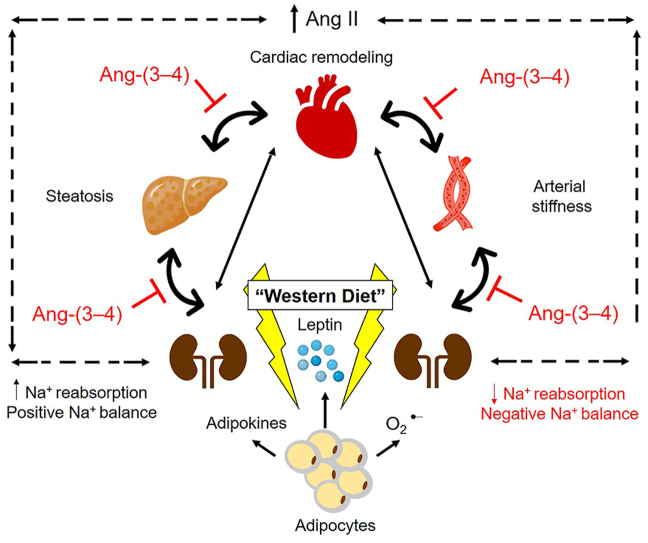

## 1 Introduction

We recently communicated [1] that young rats chronically given a hypercaloric diet in which 70% of calories come from lipids (a “Western diet” [2]) developed overweight with increased visceral fat (perirenal and epididymal), hypertension, exacerbated active Na^+^ reabsorption in kidney proximal tubule cells, and highly positive Na^+^ balance [1]. We proposed that the cardiovascular and renal alterations resulted from hyperactivation of angiotensin II type 1 receptor (AT_1_R) signaling (the Ang II→AT_1_R axis of the renin-angiotensin-aldosterone system/RAAS), which was counteracted by administration of Ang-(3–4) (Val-Tyr), the shortest renin-angiotensin-derived peptide. Ang-(3–4) antagonizes several effects of Ang II in physiological and pathological conditions [3]. One of the mechanisms for the counteracting effect is the allosteric enhancing of Ang II binding to type 2 receptors by Ang-(3–4) [4], i.e., the activation of the Ang II→AT_2_R axis of RAAS. Facing the concomitant cardiac and renal alterations, we initially proposed that the overweight resulting from chronic administration of a high-lipid (HL) diet culminates with a secondary type V cardiorenal syndrome. In this type, a systemic pathology (such as for overweight/obesity) simultaneously affects the heart and the kidney [5].

We carried out further studies in heart, kidney, and liver in adult rats, given the hyperlipidic diet from a juvenile age. The purpose was to investigate whether or not overweight/obesity provokes early structural lesions in these organs, in addition to the vascular and renal functional alterations. The driving ideas for the present study came from the central role that the visceral fat has in the activation of the Ang II→AT_1_R axis of RAAS [6] and from the hypothesis that being overweight/obese could provoke concomitant heart, kidney, and liver structural alterations as the result of upregulation of Ang II→AT_1_R signaling. The stimulus of proinflammatory cytokines by the visceral fat contributes to generalized inflammation and lipotoxicity [7,8].

## 2 Methods

### 2.1 Ethical considerations

The study was approved by the Committee for Ethical Use of Animals in Experimentation at the Federal University of Rio de Janeiro (protocol 075/19), and was carried out following the ARRIVE guidelines for preclinical animal studies.

### 2.2 Diets and animal groups

The animals were bred, maintained and studied in the Vivarium for Neglected Diseases and Malnutrition of the Carlos Chagas Filho Institute at Federal University of Rio de Janeiro. Male Wistar rats received ad libitum filtered water and a commercial diet for rodents (CTR) or a hyperlipidic diet (HL) (PRAG Solutions, São Paulo, Brazil) from 58 to 164 days of age. At day 162 part of the animals received 4 doses by gavage, at 12 h intervals, of vehicle (water) or Ang-(3–4) (80 mg/kg) (Aminotech, Diadema, Brazil), giving the groups CTR, HL, CTR + Ang-(3–4) and HL + Ang-(3–4). The total number of animals in this study was 65. A random number table was used to allocate the rats to the different groups and a single observer (M.A.E.P.) was aware of the group allocation in the different experiments. The same observer controlled the order of treatments and measurements.

### 2.3 Histological analysis of collagen deposits in heart and kidney

Small fragments of left ventricle near the apex and of renal cortex (∼50 mg) were removed immediately after euthanasia at day 164, suspended in 4% paraformaldehyde (w/v) for 24 h, embedded in paraffin and then cut in slices 4 μm thick. After staining with Picrosirius Red, the images were obtained using an Eclipse microscope (Nikon, Tokyo, Japan) coupled to an Evolution camera (Media Cybernetics, Rockville, MD, USA) and quantified using the ImageJ software (1.4.3.67 version) from the ratio, in each area, of red pixels/total pixels.

### 2.4 Blood pressure determinations

Blood pressure was measured by pletismography (model V3.0 Insight, Ribeirão Preto, Brazil) in rats aged 164 days, as previously described [1].

### 2.5 Recording of ultrasound images

Rats (aged 164 days) were anesthetized with isofluorane. Images were obtained with the high-resolution ultrasound Vevo® 2100 (FUJIFILM VisualSonics, Toronto, Canada) coupled to a 20–70 MHz as described by Marshall et al. [9] with slight modifications. Briefly, images were recorded from the right upper retroperitoneal region and the right liver lobe delimitated by the circle tool of the system. After transforming the images to the JPEG format, the hepatorenal index was calculated from the mean brightness and size in pixels from the two organs using the software ImageJ (1.4.3.67 version).

### 2.6 Statistical analysis

The samples size was calculated according to Ref. [10]. The samples followed a normal distribution. Using unpaired Student's *t*-test compared two means. Four means were compared by one-way ANOVA followed by Bonferroni's test for selected pairs.

## 3 Results

**Fig. 1:**
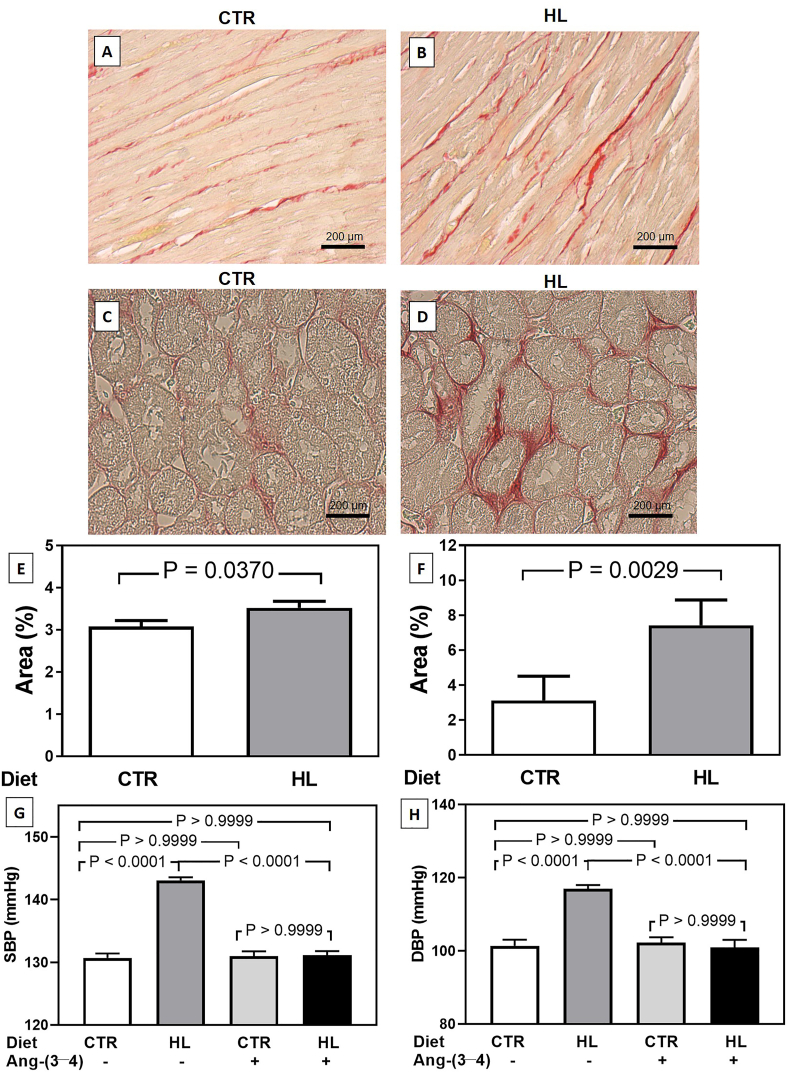
Picrosirius staining of the left ventricle and renal cortex, and systolic blood pressure in rats given control (CTR) and high-lipid content (HL) diets. (A, B) Representative images from left ventricular tissue. (C, D) Representative images from renal cortical tissue. (E, F) Graphical representation of the surface areas of the collagen network as stained by picrosirius red in cardiac and renal tissues, respectively. Left ventricle CTR: n = 4 rats; total analysed areas = 40. Left ventricle HL: n = 5 rats; total analysed areas = 50. Renal cortex CTR: n = 4 rats; total analysed areas = 40. Renal cortex HL: n = 5 rats; total analysed areas = 50. Differences were assessed using unpaired Student's *t*-test. P values are indicated within the panels. (G) Systolic blood pressure of CTR and HL rats aged 164 days that received or not Ang-(3–4) between days 162 and 164 of age. (H) Diastolic blood pressure of the same rats. Values are mean ± SEM. Differences were assessed using one-way ANOVA followed by Bonferroni's test for selected pairs. P values are indicated within the panel. CTR: n = 17; HL: n = 12; CTR + Ang-(3–4): n = 13; HL + Ang-(3–4): n = 14. The animals were the same immediately used for ultrasound studies (see Fig. 2). In one rat from this ensemble, liver position relative to kidney did not allow recording of adequate images. (For interpretation of the references to colour in this figure legend, the reader is referred to the Web version of this article.)

Body mass and blood pressure were the primary outcome as in a previous study [1]. The body mass of rats that received the HL lipid diet was higher than that of CTR at 162 days of age: 506 ± 16 g vs. 458 ± 15 g. These 10% increase in body mass indicates that they are in the middle of the way between overweight (6%) and obesity (13%) [11], with possible cardiovascular and renal comorbidities resulting from the adiposity-associated generalized inflammation [7,8]. Fig. 1A and B presents representative Picrosirius Red stained images from the left ventricle of CTR and HL rats, respectively; Fig. 1C and D presents those from the kidney cortex (CTR and HL groups, respectively). The quantification (Fig. 1E and F) demonstrates that the two organs from HL rats present augmented collagen deposits, which are more accentuated in renal tissue. Fig. 1G reveals that the HL rats developed systolic hypertension, which completely normalized after one day receiving four oral doses of Ang-(3–4). The blood pressure of CTR rats remained unmodified after Ang-(3–4). Since the high Na^+^ content of “Western diets” [2] could contribute to the development of diastolic hypertension, we also investigated whether this was the case with rats receiving the HL diet. Fig. 1H demonstrates that overweight rats also developed Ang-(3–4)-sensitive diastolic hypertension.

**Fig. 2:**
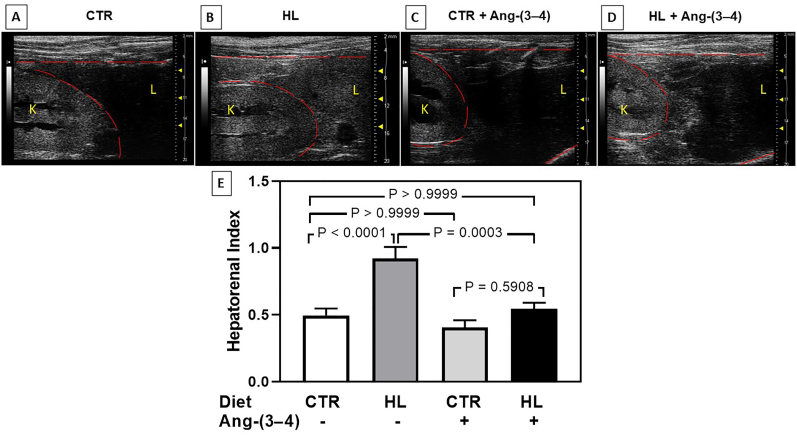
Hepatorenal index (mean hepatic brightness/mean renal brightness) in rats given control (CTR) and high-lipid content (HL) diets: effect of Ang-(3–4). (A, B, C, D) Representative abdominal ultrasound images from CTR, HL, CTR + Ang-(3–4), and HL + Ang-(3–4) rats, respectively. Ang-(3–4) was administered as described in the text. The organs are identified by their initial letters L (liver) and K (kidney), and the images were processed as described in the text. (E) Graphic representation of the pixels densities in CTR (n = 17), HL (n = 12), CTR + Ang-(3–4) (n = 12), and HL + Ang-(3–4) (n = 14) rats. Values are mean ± SEM. Using one-way ANOVA followed by Bonferroni's test for selected pairs assessed differences. P values are indicated within the panel.

The hepatorenal index is shown in Fig. 2. Comparing the representative images from panels A and B demonstrate an accentuated increase of brightness in the liver (L) from the HL rats, without change in the brightness of kidney (K). The increase reached 100%, as shown in the bargraph presented in Fig. 2E (compare the left pair of columns), revealing the presence of steatosis, which structural correlation is the percentage increase of fat deposits. Biochemically, it corresponds to ectopic deposits of triglycerides. The lesions regressed rapidly (Fig. 2C, D, and E) in rats that received Ang-(3–4); the hepatic brightness returned to the levels found in CTR rats, which were not modified by Ang-(3–4).

## 4 Discussion

The scenario of fibrosis shown in Fig. 1 reveals previous and early increased production and release of proinflammatory cytokines [12]. The production of the hepatic Fetuin-A also increases and, therefore, the inhibition of the insulin cascade and the release of inflammatory cytokines [13]. At the same time, Fetuin-A allows saturated fatty acid to stimulate the type 4 Toll-like receptors (TLR4) of macrophages [13], thus accelerating the conversion of type M2 macrophages toward the M1 phenotype [14]. At the renal sinus fat level, Fetuin-A promotes the transition from a “protector” profile to a “lesional” one, propagating and amplifying the release of proinflammatory cytokines [14]. Increased proinflammatory activity at the level of renal sinus could also be responsible for the more intense fibrosis in the renal cortex than in the left ventricle (Fig. 1A–F).

We previously demonstrated that activation of TLR4 is a central mechanism in the genesis of inflammatory cardiac lesions after acute renal injury [15], an example of type III cardiorenal syndrome [5]. Since Fetuin-A also alters the cardiac metabolism [14] and the overweight/obesity of rats receiving the HL diet is associated with hypertension and RAAS-mediated molecular alterations in renal Na^+^-transporting ATPases [1], the data from Figs. 1 and 2 allows us to propose that the Western diet HL can lead to a hepatocardiorenal syndrome, an emerging concept in pathology [16]. In terms of mechanisms of systolic blood pressure alterations, it is possible that alterations of the perivascular adipose tissue – the equivalent to the visceral adipose tissue – contributes to increased vascular tonus [17] and stiffness [18] in the aorta and mesenteric arteries through increased secretion of cytokines and adipokines [19]. Diastolic hypertension could probably be due to the higher Na^+^ content of the HL diet – when compared to the CTR diet – in combination with upregulated renal Na^+^-transporting ATPases [1].

The complete and faster regression of steatosis after administration of Ang-(3–4) also entails accentuated, rapid, and continuous lipolysis, probably resulting from the activation of both the adipose triglyceride lipase (ATGL) by cyclic AMP-dependent protein kinase (PKA) [20] and the hormone-sensitive lipase (HSL) by catecholamines [21], whose defects play a central role in obesity [22]. We propose that the activated cyclic AMP-dependent protein kinase coupled to the upregulated Ang II→AT_2_R axis [4] and these lipases antagonize the Ang II→AT_1_R→protein kinase C proinflammatory and antilipolytic signaling axis.

## 5 Conclusion

In summary, the observations communicated here provide valuable evidence regarding a hepatocardiorenal syndrome [15] induced by chronic administration of a diet with a high-lipid content, in which abnormal upregulation of the Ang-(3–4)-sensitive Ang II→AT_1_R axis of RAAS culminates with connected cardiac and renal fibrosis, liver steatosis, arterial hypertension, and augmented renal Na^+^ reabsorption [1] as the prominent pathological disturbances.

## Financial support

This work is supported by 10.13039/501100003593Brazilian Research Council/CNPq (grant 440544/2018-1), 10.13039/501100004586Rio de Janeiro State Foundation/FAPERJ (grants E−26/210.890/2019, E−26/201.909/2020 and E−26/200.866/2021), and the 10.13039/501100002322Brazilian Federal Agency for Support and Evaluation of Graduate Education/CAPES (grants 88887.320213/2019-00 and 88887.623346/2021–00).

## CRediT authorship contribution statement

**Thuany Crisóstomo:** Conceptualization, Formal analysis, Investigation, Writing – original draft, Writing – review & editing. **Marco A.E. Pardal:** Formal analysis, Investigation. **Simone A. Herdy:** Conceptualization, Formal analysis, Investigation, Writing – original draft, Writing – review & editing. **Humberto Muzi-Filho:** Conceptualization, Formal analysis, Writing – original draft, Writing – review & editing. **Debora B. Mello:** Conceptualization, Formal analysis, Investigation. **Christina M. Takiya:** Conceptualization, Formal analysis. **Rafael Luzes:** Conceptualization, Writing – original draft, Writing – review & editing. **Adalberto Vieyra:** Conceptualization, Formal analysis, Writing – original draft, Writing – review & editing, Supervision, Funding acquisition.

## Declaration of competing interest

The authors declare no conflict of interest.
